# Fetal treatment and long-term neonatal outcomes in severe maternal red cell alloimmunization – a single-centre experience

**DOI:** 10.1515/crpm-2024-0040

**Published:** 2025-06-09

**Authors:** Vita Andreja Mesarič, Irena Bricl, Erika Hrastar, Lilijana Kornhauser Cerar, Jana Lozar Krivec, Miha Rus, Derek P. de Winter, Tanja Premru Sršen

**Affiliations:** Department of Perinatology, Division of Gynaecology and Obstetrics, 37667 University Medical Centre Ljubljana, Ljubljana, Slovenia; Faculty of Medicine, University of Ljubljana, Ljubljana, Slovenia; Blood Transfusion Centre of Slovenija, Ljubljana, Slovenia; Neonatology Section, Department of Perinatology, Division of Gynaecology and Obstetrics, University Medical Centre Ljubljana, Ljubljana, Slovenia; Department of Neonatology, University Children’s Hospital, University Medical Centre Ljubljana, Ljubljana, Slovenia; Department of Pediatrics, Division of Neonatology, Willem-Alexander Children’s Hospital, Leiden University Medical Center, Leiden, The Netherlands; Department of Immunohematology Diagnostic Services, Sanquin Diagnostic Services, Amsterdam, The Netherlands

**Keywords:** haemolytic disease of the fetus and the newborn, severe maternal alloimmunization, intrauterine transfusion, neurodevelopmental outcome

## Abstract

**Objectives:**

Haemolytic disease of the fetus and newborn (HDFN) occurs due to maternal IgG alloantibodies that actively cross the placenta and bind to paternally derived fetal antigens on the erythrocytes. The aims of this study were to describe the Slovenian cohort of patients with severe HDFN, who required fetal treatment, to review the fetal treatment strategies, and to describe pregnancy and neurodevelopmental outcomes.

**Case series presentation:**

Data on patients who developed severe HDFN between 2006 and 2021 and were treated at our institution were collected retrospectively. Primary care pediatricians were contacted regarding neurodevelopmental outcomes of surviving infants. There were 19 pregnancies affected with severe HDFN. The most commonly implicated antigen was RhD. Seventeen children were liveborn. Sixteen fetuses were treated with intrauterine transfusion (IUT). Two children had developmental delay at the corrected age of 2 years.

**Conclusions:**

In this study, the Slovenian national cohort of severe cases of HDFN is described for the first time. Prevalence of RhD alloimmunization was higher in comparison to the literature. A combined treatment with therapeutic plasmapheresis, immunoglobulins and IUT was successful. Three quarters of newborns were born in the late preterm period. Overall survival rate and long-term neonatal adverse outcomes in our cohort were in line with the literature.

## Introduction

Haemolytic disease of the fetus and the newborn (HDFN) occurs due to maternal immunoglobulin G (Ig G) alloantibodies that actively cross the placenta and bind to paternally derived fetal antigens on the erythrocytes. The most commonly implicated antigens are D, followed by K, c and E [1]. Antibody-coated erythrocytes are haemolyzed in the fetal spleen potentially resulting in fetal anemia. Compensatory erythropoiesis is stimulated and fetal cardiac output increases in response to hypoxemia [2]. The fetal circulation becomes hyperdynamic, which can lead to the development of cardiomegaly, hydrops and finally intrauterine fetal demise (IUFD) can occur without timely detection and treatment [3]. After birth, the immature neonatal liver is unable to conjugate increased levels of bilirubin, which is deposited in the neonatal tissues, including the central nervous system, particularly the basal ganglia and brainstem nuclei, potentially leading to bilirubin encephalopathy. Chronic bilirubin encephalopathy involves psychomotor handicap, athetoid cerebral palsy and hearing problems and is an important cause of severe long-term morbidity [4].

Even though no specific cure for HDFN exists, its incidence, especially in high-income countries, has dramatically decreased due to preventative efforts such as screening of all pregnancies for RhD status, subsequent Rhesus immunoprophylaxis administration and transfusions of RhD matched and Kell negative blood products to women of child-bearing age. The rate of RhD alloimmunization is currently 0.3–0.6 % in developed countries [5]. Nevertheless, HDFN has not yet been eradicated.

Owing to the improvement of fetal and postnatal management strategies, the outcome of affected pregnancies is generally good. Through enhanced surveillance of high-risk pregnancies we can timely detect fetal anemia [6]. Standard therapy for fetal anemia is intrauterine transfusion (IUT) which improves fetal haemoglobin levels, reverts hydrops and prolongues the duration of pregnancy, and consequently markedly improves the outcome [7], [8], [9]. In severe maternal red blood cell (RBC) alloimmunization, several adjunctive fetal therapy strategies exist to postpone the need for IUT beyond 20 gestational weeks such as intravenous immunoglobulins (IVIg) alone or in combination with therapeutic plasmapheresis (TPE) [10], [11], [12]. After birth hyperbilirubinemia may be treated with phototherapy and in severe cases with exchange transfusions (ET). Anemia due to haemolysis or inhibition of erythropoiesis may be treated with red blood cell transfusions or erythropoietin [13]. Phototherapy is of extreme importance to diminish the possibility of bilirubin encephalopathy [14].

Due to the rarity of HDFN, and numerous treatment options, it is crucial to evaluate and report upon the management of affected pregnancies and newborns. We therefore aimed to describe the Slovenian cohort of patients with HDFN, who required fetal treatment, to review fetal treatment strategies and to describe pregnancy and long-term neurodevelopmental outcomes.

## Materials and methods

This retrospective single-centre cohort study was conducted at the Department of Perinatology of the University Medical Centre (UMC) Ljubljana, which is the referral institution for the management of the most complicated perinatal pathologies, including HDFN. Slovenia has a population of two million and approximately 18,000 children are born annually [15].

Our institution was one of the 31 centres from 22 countries that have taken part in a worldwide multicentric cohort study DIONYSUS (worlDwIde cOllaboratioN for hemolYtic diSease of the fetUs and newborn) on antenatal and postnatal management practice variations in HDFN between 2006 and 2021.

Perinatal and neonatal data were collected from the medical records: hospital’s informational system Hipokrat© and archived medical files. Data regarding type of alloimmunization, titre and ADCC values were provided by the Blood Transfusion Centre of Slovenia. Primary care paediatricians were contacted by a member of our team to gather information about neurodevelopmental progress at the corrected age of two years. The tests performed at the corrected age of two years were not standardised as no specific guidelines exist.

Maternal information included demographics, previous pregnancy outcomes, and RBC transfusion data (blood group, antibody specificity, serological titre, ADCC values, previous transfusions). For women who appeared more than once in the database, each alloimmunized pregnancy was recorded as a separate event.

Antenatal data included gestational age at the first detection of antibodies, type of antibodies, titres, ADCC values, abnormal ultrasound findings, type of fetal treatment, gestational age at the start and stop of fetal treatment, IUT characteristics, fetal laboratory values before and after each IUT, complications following IUTs. Neonatal data included timing and mode of delivery, birth weight, Apgar scores, laboratory results, postnatal management strategies.

Outcome measures included fetal and neonatal survival, complications of fetal treatment, adverse neonatal outcomes, and neurodevelopmental status at the corrected age of 2 years. Adverse neonatal outcomes included the need for respiratory support and severe neonatal morbidity, defined as sepsis with a positive blood culture, necrotising enterocolitis (NEC) ≥Bell stage 2A and the presence of severe cerebral injury (SCI). SCI was defined as periventricular leukomalacia (PVL) grade 2 or more, intraventricular haemorrhage (IVH) grade 3 or more, ventriculomegaly, arterial, or venous infarct or presence of porencephalic or parenchymal cysts.

No national guidelines exist on the management of DHDFN in Slovenia. Until 2023 the management was guided by the American Academy of Pediatrics (AAP). Currently, neonatologists are following the National Institute for Health and Care Excellence (NICE) Guidelines. IVIg are not used routinely and are used in rare circumstance when ET is not possible, such as inability to transport the newborn into the referral center or lack of blood products. Newborns undergo Auditory Brainstem Response testing and if deemed pathological, Brainstem Evoked Auditory Potential Testing is also performed. Children are followed up in the outpatient developmental clinics for at least 2 years. 

### Statistical analysis

All data was aggregated in the Castor© Clinical Data Platform. Statistical analyses were performed using SPSS software (version 25.0). Continuous data is presented as median (range) and categorical data as proportions (n/N, %).

### Ethical considerations

The study was performed in line with the principles of the Declaration of Helsinki and was approved by Medical Ethics Committee of the Republic of Slovenia (0120–554/2021/8). Informed consent for data collection and publication was obtained from the patients.

## Case series presentation

### Pregnancy characteristics

A total of 18 women with 19 pregnancies affected by severe HDFN that required fetal treatment in the 16-year period between January 2006 and December 2021 were identified and included. All pregnancies were singleton and conception was spontaneous. Seventeen children were liveborn. Two pregnancies (11.1 %) resulted in IUFD. The first pregnancy that ended in IUFD was complicated by severe anemia due to posttransfusion anti-D alloimmunisation, which we attempted to correct with several consecutive IUTs every 2 days. After the third IUT at 29 4/7 a sudden cardiac arrest occured. The second pregnancy was affected by Kell alloimmunisation, which was treated with three IUTs. After each IUT the fetus developed transient bradycardia with spontaneous resolution. The IUFD occurred several hours after the third IUT at 30 4/7.

Five women had a history of an IUFD due to severe fetal anemia. Four out of five cases of IUFD occurred before 2006 and are therefore not included in this study.

### Alloimmunization details

The included patients were referred to our institution at a median gestational age of 8 weeks (range 6–28). The majority was referred due to a positive indirect Coombs test (ICT) (16/19, 84.2 %), whereas three cases were referred due to a history of alloimmunization. Types of immunization and pregnancy outcomes can be found in Table 1.

**Table 1:** Types of alloimmunization.

Type of alloimmunization	Number of pregnancies	Number of liveborn children	Number of IUFD
RhD	3	3	
RhD, RhC	7	6	1
RhD, RhC, RhE	1	1	
RhD, RhC, Fy^a^	1	1	
RhC, RhE, Kpa, G, Kna	1	1	
K1 (Kell)	4	3	1
RhE	1	1	
M	1	1	
Sum	19	17	2

IUFD, intrauterine fetal demise.

The event that triggered immunization was unknown in 66.7 % (12/18) of women. The known causes of immunization included inadequate prophylaxis (4/6, 66.7 %), early pregnancy bleeding (1/6, 16.6 %) and one woman who received RhD positive blood products (1/6, 16.6 %).

The median value of peak titres recorded during the pregnancies was 512 (range 128–4,096) for RhD HDFN and 256 (range 2–1,024) for Kell HDFN. In 94.7 % (18/19) an ADCC was performed. Median value of expected haemolysis was 72.5 % (range 10–80).

### Fetal management strategies of HDFN

A total of 18 fetuses (18/19, 94.7 %) received at least one IUT. Among those, we managed two cases (2/19, 10.5 %) with a combination of IUT, IVIg and TPE. The remaining case (1/19, 5.3 %) was treated with IVIg application only.

One mother was treated with a combination of methods due to a history of severe HDFN in the previous pregnancy, which ended in IUFD during IUT due to the sudden cardiac arrest at 29 4/7 despite having received two IUTs previously. She had anti-D, anti-C and anti-E antibodies. Highest titre of antibodies (anti-D) was 1,024 with the highest ADCC of 80 %. She received 9 IVIg treatments between 10 and 19 weeks of gestation, followed by 9 TPEs between 20 and 23 weeks and 9 IUTs between 21 and 34 weeks. The mother underwent induction of labour and vaginally delivered a healthy girl at 36 3/7 weighing 2660 g with an Apgar score of 9 at 5 min. The newborn received one RBC transfusion in the first week of life and was discharged on day 5 without sequelae, however readmission was required at day 28 of life for another RBC transfusion. Her development at the corrected age of 2 years was normal.

The second patient treated with a combination of IUT, IVIg and TPE had Kell alloimmunization with the highest titre of 256 and ADCC 35 %. She received 15 IVIg treatments between 15 and 25 weeks of gestation due to the high titre of Kell antibodies (256) and ADCC (25 %) in the first trimester, followed by 3 TPE from 25 weeks on and 2 IUTs at 26 1/7 and 30 5/7. Primary caesarean section was performed at 33 5/7 after two doses of betamethasone for lung maturation due to the need for the third IUT, which the mother decided against. A healthy girl was born with a birthweight of 1890 g and Apgar score of 9 at 5 min. She required 4 days of intensive phototherapy and non-invasive respiratory support and was discharged on day 21. Her development at the corrected age of 1 year was normal.

The patient, treated with IVIg only, was alloimmunized to RhD and RhC and the cause of alloimmunization was unknown. This was her fourth pregnancy. The previous pregnancy ended in fetal death of a chromosomally normal fetus at 18 weeks of gestation with the titre of anti-D antibodies 32 and ADCC 60 % in the first trimester. No other cause of death was identified. The highest titre of anti-D antibodies in the fourth pregnancy was 256 and the highest ADCC was 55 %. After fetal genotyping, she received five IVIg applications between 18 and 22 weeks of gestation and vaginally delivered a healthy boy at 36 weeks and 3 days. The newborn required 2 days of intensive phototherapy, one ET and non-invasive respiratory support and was discharged home after 15 days. He had no neurodevelopmental delay at the age of 2 years.

### Characteristics of IUTs

The characteristics of IUTs are shown in Table 2. We performed 71 IUTs in 18 pregnancies in the observed period with a median of 2.4 (range 1–9) IUTs per pregnancy. The most common indication for IUT was elevated MCA PSV (27/71, 38.0). Most common access for IUT was the umbilical cord at the insertion into the placenta (47/71, 66.2 %). Complications occurred in 7 % (5/71) of the IUTs. Median value of the haemoglobin level before the IUT was 90 g/L (range 11–128), values of other parameters are shown in Table 2.

**Table 2:** Characteristics of IUTs.

IUT (n=71 in 18 pregnancies)	n (%) or median (range)
Pregnancy outcome – Live birth	16 (88.9)
– IUFD	2 (11.1)
Number of IUTs per pregnancy	2.4 (1–9)
Indication for IUT – MCA PSV>1.5MoM	27 (38.0)
– Scheduled after previous IUT	14 (19.7)
– MCA PSV>1.5MoM and scheduled	8 (11.3)
– Anemia	9 (12.7)
– Hydrops	4 (2 different pregnancies) (5.6)
– High titres/ADCC	3 (4.2)
– Unspecified	6 (8.5)
Access – Umbilical cord at the insertion into the placenta	47 (66.2)
– Intraabdominal portion of the umbilical vein	1 (1.4)
– Umbilical cord free loop	2 (2.8)
– Unspecified	21 (29.6)
Hb prior to IUT (g/L)	90 (11–128)
Ht prior to IUT (/)	0.278 (0.036–0.83)
Ret prior IUT ( × 10^9^/L)	227 (11–496)
Hb after IUT (g/L)	144 (63–212)
Ht after IUT (/)	0.429 (0.176–0.67)
Complications – IUFD	2
– Fetal bradycardia	2
– PPROM	1

IUFD, intrauterine fetal demise; IUT, intrauterine transfusion; MCA PSV, middle cerebral artery peak systolic velocity; ADCC, antibody-dependent cell-mediated cytotoxicity; Hb, hemoglobin; Ht, hematocrit; Ret, reticulocyte count; PPROM, preterm prelabour rupture of membranes. Data is presented as n (%) or median (range).

### Delivery and neonatal outcome

There were 17 live births, 16 following fetal treatment with IUT and one after fetal treatment with IVIg only. No neonatal deaths occurred; hence the overall survival rate was 89.5 %.

Characteristics of liveborn children treated with IUT are shown in Table 3. The majority was born with primary caesarean section (11/16, 68.8 %), followed by three vaginal births (3/16, 18.8 %) and two secondary caesarean sections (2/16, 12.4 %). Median gestational age at delivery was 34 weeks (range 30 0/7–36 4/7) with a median birthweight of 2,310 g (range 1,440–2,960 g). Median haemoglobin at birth was 122.5 g/L (range 70–169) and median total bilirubin 111 μmol/L (range 63–270). All 16 liveborn newborns were admitted to the neonatal intensive care unit (NICU) and median hospital stay was 11 days (range 5–44). Eleven (11/16, 68.8 %) newborns required phototherapy with a median duration of 2 days (range 1–5). A quarter (4/16, 25.0 %) had one ET and 2 (12.4 %) required a transfusion of RBC within the first week of life. Nine (56.3 %) were treated with erythropoietin. There was one case with suspected acute bilirubin encephalopathy with slowed conduction at the auditory brainstem responses (ABR) and right-sided hearing loss. There were no cases of NEC, sepsis or SCI. Majority of the newborns did not require any respiratory support (9/16, 56.2 %), six (6/16, 37.5 %) required non-invasive respiratory support and one (1/16, 6.3 %) mechanical ventilation.

**Table 3:** Characteristics of liveborn infants treated with IUT (n=16).

Characteristic	n (%) or median (range)
Mode of delivery – Vaginal	3 (18.8)
– Primary caesarean section	11 (68.8)
– Secondary caesarean section	2 (12.4)
Gestational age at delivery, weeks	34.0 (30 0/7–36 4/7)
Gestational age at delivery – <32 weeks	3 (18.8)
− 32–34 weeks	1 (6.3)
− 34–37 weeks	12 (74.9)
– >37 weeks	0 (0.0)
Birth weight, g	2310 g (1,440–2,960)
Apgar score at 5 min	9 (6–9)
Apgar score <7 at 5 min	1 (6.3 %)
Hydrops at delivery	0 (0.0 %)
Hemoglobin at birth (g/L)	122.5 (70–169)
Total bilirubin at birth (µmol/L)	111 (63–270)
NICU admission	16 (100.0)
Hospital stay, days	10 (5–44)
Neonatal treatment for HDFN required	15 (93.8)
Phototherapy	11 (68.8)
Duration of phototherapy, days	2 days (1–5)
Exchange transfusion	4 (25.0)
Transfusion within 1 week	2 (12.4)
Erythropoietin	9 (56.3)
Possible bilirubin encephalopathy	1 (6.3)
Congenital anomalies	1 VSD, 1 ASD (12.6)
Severe neonatal morbidity (NEC, sepsis, SCI)	0 (0.0)
Respiratory support – None	9 (56.2)
– Non-invasive	6 (37.5)
– Mechanical ventilation	1 (6.3)
Neonatal deaths	0 (0.0)
Development at the corrected age of 2 years	11 (68.8) normal
	1 (6.3) not applicable
	1 (6.3) moderate developmental delay
	1 (6.3) severe developmental delay
	2 (12.6) lost to follow-up

NICU, neonatal intensive care unit; NEC, necrotising enterocolitis; RDS, respiratory distress syndrome; PVL, periventricular leukomalacia; IVH, intraventricular hemorrhage; SCI, severe cerebral injury. Data is presented as n (%) or median (range).

### Neurodevelopmental outcome

Eleven children (11/16, 68.8 %) had a normal development at the corrected age of 2 years, two (2/16, 12.6 %) were lost to follow-up, one (1/16, 6.3 %) was younger than two years at the time of data collection, her development at the corrected age of 1 year was normal. Two children (2/16, 12.6 %) had developmental delay, one moderate and one severe according to the Denver Developmental Screening Test II or routine psychological testing at discretion of their primary care paediatrician. We looked closely at the two children with the developmental delay at the corrected age of 2 years. Data is shown in Table 4.

**Table 4:** Infants with developmental delay after IUT for severe anemia.

Case	1 (severe delay)	2 (moderate delay)
Sex	Male	Female
Sensibilization type	RhD	RhE
Highest titre	512	64
ADCC	>80 %	10 %
Hydrops	No	No
Number of IUT	1	2
Hb at 1st IUT	83 g/L	No data
Additional prenatal treatment	None	None
Mode of delivery	Vaginal	Primary caesarean section
GA at birth	30+5	33+0
Birthweight	1440 g	1560 g
Apgar at 5 min	9	9
Received steroids (GA, weeks)	No (na)	Yes (28 5/7)
Hb at birth	122 g/L	123 g/L
Total bilirubin at birth	270 μmol/L	No data
Severe neonatal morbidity	No	No
Respiratory support	Non-invasive	No
Phototherapy, days	Yes (3)	Yes (1)
RBC transfusion	No	No
Exchange transfusion	Yes	No
Bilirubin encephalopathy	Possible	No
Congenital abnormalities	ASD	No
Special needs education	Yes	No

ADCC, antibody-dependent cell-mediated cytotoxicity; IUT, intrauterine transfusion; Hb, hemoglobin; GA, gestational age; RBC, red blood cell.

A boy with severe developmental delay requiring special needs education was born vaginally at 30 5/7 following PPROM and spontaneous contractions with a birthweight of 1440 g. Apgar score at 5 min was 9. Haemoglobin after birth was 122 g/L and total bilirubin 270 μmol/L and the maximum value was 302 on day 2 of life. He required non-invasive respiratory support, one ET, and 3 days of phototherapy. His mother was alloimmunized to RhD and peak titre during pregnancy was 512 with the highest ADCC result of >80 %. During pregnancy one IUT at gestational age 30 1/7 was performed, haemoglobin at the time of the IUT was 83 g/L. After birth, he was diagnosed with an atrial septum defect (ASD) and slowed conduction in the hearing nerves with right-sided hearing loss (possible bilirubin encephalopathy). Cranial ultrasound was reported to be within the normal range, brain magnetic resonance imaging was however not performed.

A girl was born at 33 0/7 to a mother who was treated due to RhE alloimmunization. Highest titre was 64 with an ADCC of 10 %. She received 2 IUTs at gestational ages 28 6/7 and 30 4/7. A primary caesarean section was performed at 33 0/7, her birthweight was 1560 g, Apgar score at 5 min was 9. Haemoglobin at birth was 123 g/L. She required 1 day of phototherapy. She can attend typical nursery school, where she receives help from a learning assistant.

## Discussion

This is the first study describing severe cases of HDFN requiring fetal treatment at our institution, which is the only fetal therapy centre in Slovenia. This article includes all cases of severe HDFN over a 16-year period across the country and describes the fetal and postnatal management at a national level.

Despite the wide-spread use of anti-D immunoglobulins for the prevention of alloimmunization in our country, by far the most commonly implicated antigen in severe HDFN was RhD (12/16, 75.0 %). Like in our study, RhD was identified as the causative antigen in 71.6 % of cases reported by Pan et al. [16]. Our results highlight the importance of avoiding transfusion mistakes as well as the importance of prophylactic use of the anti-D immunoglobulins in case of potentially sensitising events which should never be forgotten.

Several fetal therapy centres alternatively use IVIg and TPE in severe maternal RBC alloimmunization to delay the onset of fetal anemia, for example high antibody titres, high ADCC values, or in mothers with unfavourable obstetric history (such as IUFD due to HDFN in the previous pregnancy). In the largest study from the 1990s, starting IVIg therapy before 21 weeks of gestation, followed by IUTs after 20 weeks appeared to reduce fetal mortality from 20 to 15 % [17]. In a multicentre PETIT study, IVIg was associated with a reduced risk of hydrops and the need for ET compared with the untreated group [10]. IVIg treatment, when started before 13 weeks of gestation, demonstrated a potential to delay the need for IUT in comparison to the previous pregnancy. However, the overall infant survival in the PETIT study was high, 88 %, but did not differ between the IVIg-treated and untreated groups. As in our cases, a combined treatment with TPE and IVIg early in pregnancy and IUT later in pregnancy, if needed, was reported to be successful in the management of severe maternal RBC alloimmunization in case reports [11], 18], 19].

One of our patients with RhD and RhC alloimmunization received 5 doses of IVIg from 18 weeks until 23 weeks without the need for subsequent IUTs and a healthy boy was born at 36 3/7 without sequelae, which highlights the potential efficacy of IVIg. The decision for the use of IVIg was based on the high titres and ADCC in the first trimester and a history of fetal death at 18 weeks of pregnancy in her previous pregnancy (her third and the second pregnancy with alloimmunization) with no cause of death identified. In another case we opted for IVIg applications starting from week 10 due to previous IUFD of a severely anemic fetus at 29+4 weeks due to RhD alloimmunization as a consequence of RhD positive blood products. After 20 weeks we continued with TPEs and 9 IUTs. A healthy girl was born vaginally at 36 3/7 and has no neurodevelopmental delay at the age of 4 years. Despite promising results of alternative treatment options of severe maternal RBC alloimmunization from retrospective studies and case reports, we still lack evidence from randomized controlled trials to prove the efficacy of IVIg and TPE and the decision for alternative fetal treatment options remains individualized.

Among 71 IUTs performed in our institution in the observed period, the rate of severe adverse events was 7 %, which is comparable to larger centres. Importantly, there were no cases of bleeding from the puncture site or infections and only one case of PPROM, followed by a preterm delivery, which are commonly described complications of IUT [16], 20].

Outcomes of the fetal treatment for severe HDFN in our cohort are favourable. The overall survival rate was 89.5 %. No neonatal deaths occurred, which is in line with the literature [20]. Neonatal outcomes in our cohort are also comparable to international data. All but one newborn had an Apgar score of more than 7 after 5 min and none of them was hydropic at the time of delivery. Most children were born in the late preterm period (75.0 %), unfortunately, none were born after 37 weeks of gestation. According to a systematic review by de Winter et al. [2], most children with HDFN are born in the late preterm period, around 35 weeks’ gestation. In the future we should avoid delivering neonates in the late preterm period and aim for delivery after 37 weeks with the last IUT being around 35 weeks. Late preterm babies are at higher risk of complications than full-term babies, such as respiratory distress, temperature instability, hypoglycaemia, jaudnice, feeding difficulties, apnea, sepsis and they carry and increased risk of long-term morbidities, such as neurodevelopmental delay, cerebral palsy, chronic respiratory and metabolic disease [21].

Most newborns received the postnatal care for HDFN (93.8 %), with only four cases requiring ET (25.0 %), which is in line with literature data and is not a reflection of unsatisfactory fetal management [16]. There were no neonatal deaths in our cohort, highlighting quality postnatal care at our institution.

Neurodevelopmental outcome is often lacking in research on rare diseases. In this study, we were able to assess it. One child was classified as having moderate neurodevelopmental delay at the corrected age of 2 years without the requirement of special needs education. Interestingly, the sensitising antigen was RhE, which has been described as a rare cause for severe HDFN [22] and the highest titre was 64 with ADCC of 10 %. She was, however, born prematurely and was small for gestational age, which are both known risk factors for developmental delay [23], 24]. One of the children from our cohort had severe neurodevelopmental delay and has special needs. He was born prematurely at 30 5/7 and the LOTUS study has clearly shown that prematurity is a significant risk factor for neurodevelopmental delay in HDFN [25]. The total bilirubin level at birth was high, 270 μmol/L, and he required ET. Data on acute bilirubin-induced neurological dysfunction are missing from the medical records, and brain MRI was not performed, therefore bilirubin toxicity cannot be excluded. Overall rate of adverse long-term neonatal outcomes in our study was 11.8 %, consistent with the rate reported in the literature and it is likely that the neurodevelopmental delay is due to prematurity rather than severity of HDFN [16], 25].

### Limitations

Our study is limited by a small sample size, therefore a comparison of subgroups according to sensitising antigen is impossible. Severe HDFN is a rare disease and even though we have studied the entire Slovenian population of pregnant women requiring fetal treatment for HDFN, the number of cases is small. Nevertheless, the study describes the fetal management strategies and outcomes on a national level. Combining cases from different centres in a large multicentre study such as DIONYSUS will provide better insight into the management of HDFN and long-term maternal and neonatal outcomes. The data was collected retrospectively, which may lead to underreporting adverse events and missing data, however prospective data collection may be unfeasible. The follow-up for neurodevelopmental delay was not done with standardized tests or questionnaires as it was performed by different primary care paediatricians in different institutions.

## Conclusions

In conclusion, HDFN is not obsolete, and awareness of its potential severity in affected pregnancies is essential. Cases of severe HDFN may have excellent outcomes if the pregnancies receive multidisciplinary and timely management. Referral should be made early in the course of the disease, which is enabled by the nationwide screening programs. Structured fetal monitoring is essential and a timely decision for fetal therapy must be made to achieve the best outcomes. As most newborns will require postnatal care, they should be born in a specialized unit with a high-quality NICU.
